# The Sequence and Three-Dimensional Structure Characterization of Snake Venom Phospholipases B

**DOI:** 10.3389/fmolb.2020.00175

**Published:** 2020-08-05

**Authors:** Anwar Ullah, Rehana Masood

**Affiliations:** ^1^Department of Biosciences, COMSATS University Islamabad, Islamabad, Pakistan; ^2^Department of Biochemistry, Shaheed Benazir Bhutto Women University Peshawar, Peshawar, Pakistan

**Keywords:** snake venom phospholipases B, sequence and three-dimensional structure analysis, glycosylation, structural comparison, structure-based substrate specificity and maturation

## Abstract

Snake venom phospholipases B (SVPLBs) are the least studied enzymes. They constitute about 1% of *Bothrops* crude venoms, however, in other snake venoms, it is present in less than 1%. These enzymes are considered the most potent hemolytic agent in the venom. Currently, no structural information is available about these enzymes from snake venom. To better understand its three-dimensional structure and mechanisms of envenomation, the current work describes the first model-based structure report of this enzyme from *Bothrops moojeni* venom named as *B. moojeni* phospholipase B (PLB_*Bm*). The structure model of PLB_*Bm* was generated using model building software like I-TESSER, MODELLER 9v19, and Swiss-Model. The build PLB_*Bm* model was validated using validation tools (PROCHECK, ERRAT, and Verif3D). The analysis of the PLB_*Bm* modeled structure indicates that it contains 491 amino acid residues that form a well-defined four-layer αββα sandwich core and has a typical fold of the N-terminal nucleophile aminohydrolase (Ntn-hydrolase). The overall structure of PLB_*Bm* contains 18 β-strands and 17 α-helices with many connecting loops. The structure divides into two chains (A and B) after maturation. The A chain is smaller and contains 207 amino acid residues, whereas the B chain is larger and contains 266 amino acid residues. The sequence and structural comparison among homologous snake venom, bacterial, and mammals PLBs indicate that differences in the length and sequence composition may confer variable substrate specificity to these enzymes. Moreover, the surface charge distribution, average volume, and depth of the active site cavity also vary in these enzymes. The present work will provide more information about the structure–function relationship and mechanism of action of these enzymes in snakebite envenomation.

## Introduction

Phospholipases B (PLBs) or lysophospholipases (EC3.1.1.5) are high-molecular-mass enzymes that break ester linkages of glycerophospholipids of membranes at both positions *sn-1* and *sn-2* (Shiloah et al., 1973; Rokyta et al., 2011; Chapeaurouge et al., 2015). These enzymes have been named as PLBs (Doery and Pearson, 1964), phospholipase B-like (Doery and Pearson, 1964; Aird et al., 2013), lysophospholipases (Takasaki and Tamiya, 1982), and Ntn-hydrolases (Oinonen and Rouvinen, 2000). These are reported to exist in the venom proteomes of various snakes, bee, scorpions, and insects), fungi, bacteria, animal tissues, and rice bran (Table 1).

**TABLE 1:** Occurrence of PLBs in various organisms.

Snakes	References
*Calloselasma rhodostoma, Trimeresurus insularis*, *Porthidium porrasi*, *Hypnale hypnale*, *Crotalus durissus collilineatus, Echis carinatus carinatus*, *Bothrops moojeni*, coral snake, *Naja kaouthia*, *Tropidolaemus wagleri*, Russian Vipers of Pelias Group, *Lachesis muta rhombeata*, *Porthidium lansbergii lansbergii*, *Pseudechis guttatus*, *Austrelaps superbus*, *Ovophis okinavensis*, *Protobothrops flavoviridis*, *Bothropoides jararaca*, *Bothropoides neuwiedi*, *Rhinocerophis alternatus*, *Rhinocerophis cotiara*, *Bothrops jararacussu* and *Bothrops atrox*, *Drysdalia coronoides*, *Pseudechis colletti*	Bernheimer et al., 1987; Aird et al., 2013, 2017; Marcon et al., 2013; Sousa et al., 2013; Viala et al., 2014; Jiménez-Charris et al., 2015; Wiezel et al., 2015; Kovalchuk et al., 2016; Tang et al., 2016, 2019; Zainal Abidin et al., 2016; Amorim et al., 2017; Patra et al., 2017; Tan et al., 2017; Vanuopadath et al., 2018; Jones et al., 2019; Méndez et al., 2019; Oliveira et al., 2019
**Scorpion**	
Egyptian scorpion	Doery and Pearson, 1964; Mohamed et al., 1969
**Insects**	
*Musca domestica* L., Culex pipiens fatigans	Khan and Hodgson, 1967; Rao and Subrahmanyam, 1969
**Fungi**	
*Penicillium notatum*	Fairbairn, 1948; Saito, 2014
**Bacteria**	
*Streptomyces* sp. strain NA684,	Doery and Pearson, 1964; Matsumoto et al., 2013
**Mammals**	
Bovine lysosomal phospholipase B-like protein	Repo et al., 2014
Rice bran	Contardi and Ercoli, 1933

*PLBs, phospholipases B.*

Currently, little is known about the pathological and physiological effects of these enzymes in snake venom (Rokyta et al., 2011; Chapeaurouge et al., 2015; Oliveira et al., 2019; Tang et al., 2019). Upon snakebite envenomation, snake venom PLBs (SVPLBs) display strong hemolytic and cytotoxic activities and cause myoglobinuria and cytotoxicity (Takasaki and Tamiya, 1982; Bernheimer et al., 1986, 1987). The hemolytic activity of these enzymes is related to the hydrolysis of phosphatidylcholine (Bernheimer et al., 1986).

The relative abundance of PLBs varies in snake venoms, and generally, it constitutes a small percentage of the crude venoms; for example, PLBs constitute about 0.34% of the crude venom of Elapidae (Margres et al., 2013) and in the Viperidae, this percentage varies from 0.23 to 2.5 (Sousa et al., 2013). In *Botrops* species, the highest percentage has been reported in *Rhinocerophis cotiara* (2.5% of the crude venom) (Sousa et al., 2013).

SVPLBs are high-molecular-mass proteins (∼55 kDa) (Rokyta et al., 2011; Chapeaurouge et al., 2015; Wiezel et al., 2015) with a *p*I of 6.2 (Bernheimer et al., 1987). These enzymes display maximum catalytic activity in the pH range from 8.5 to 10 (Doery and Pearson, 1964).

Research about the SVPLBs is in nascent stage, and the first sequence report (based on transcriptomic analysis) about this protein came out in 2011 (Chatrath et al., 2011; Rokyta et al., 2011). The primary structure of SVPLB contains 553 amino acids in which the first 36 amino acids form the signal peptides and the remaining 526 make the PLB domain (Rokyta et al., 2011). Both monomeric and dimeric forms of PLBs have been reported to exist in snake venoms (Bernheimer et al., 1987; Chatrath et al., 2011). SVPLBs are stable proteins and show full enzymatic activity in the temperature range of 0–60°C; however, some of these retain 47% of the biological activity even at a temperature of 100°C (Bernheimer et al., 1987).

Although the primary amino acid sequence of PLBs from a number of snake venoms is present in the protein sequence database (UniProt databank), there is no report about their three-dimensional (3D) structure. Owing to this, it is difficult to co-relate their structural properties with the function. Keeping this in view, the current work reports model-based structural characterization of PLBs from *Bothrops moojeni* venom.

## Results and Discussion

### Sequence Alignment Analysis

**FIGURE 1:**
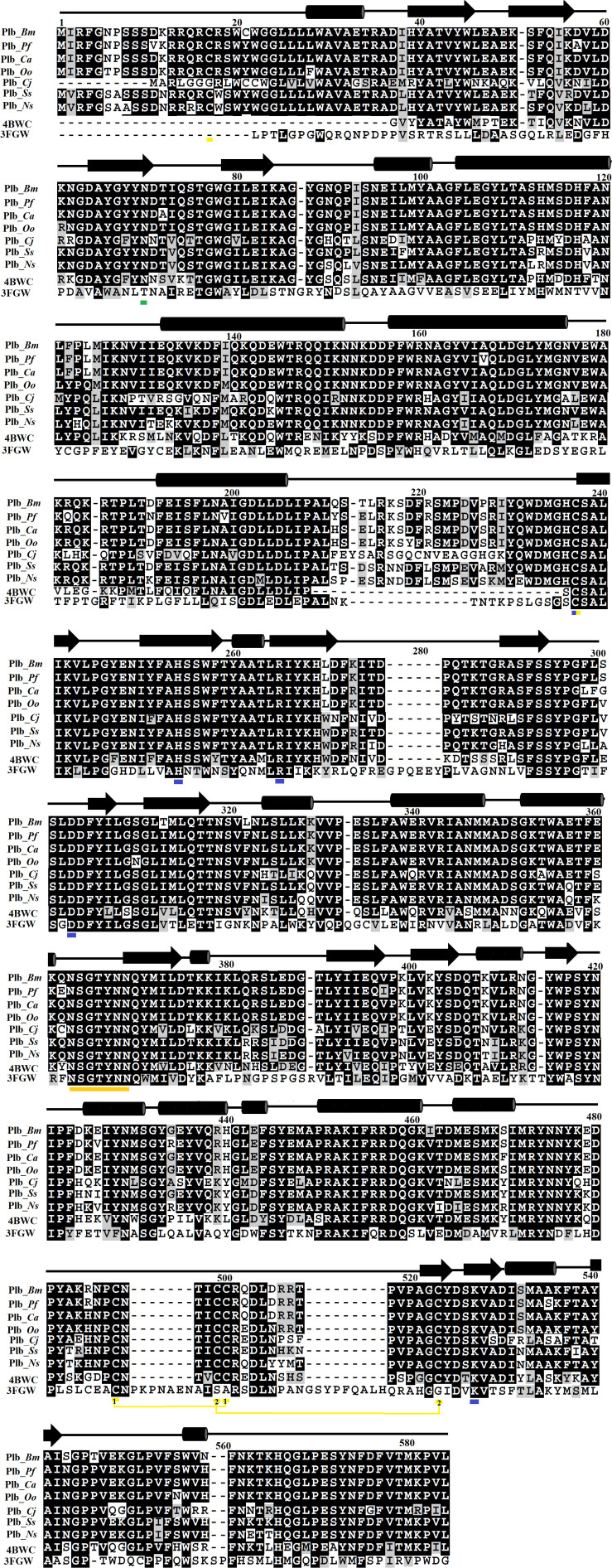
Sequence alignment among snake venom phospholipases B (SVPLBs), PLB from bovine kidneys, and 66.3 kDa protein from *Mus musculus*. PLB_*Bm*, phospholipase B from *Bothrops moojeni*; PLB_*Pf*, phospholipase B from *Protobothrops flavoviridis*; PLB_*Ca*, phospholipase B from *Crotalus atrox*; PLB_*Oo*, phospholipase B from *Ovophis okinavensis*; PLB_*Cj*, phospholipase B from *Coturnix japonica*; PLB_*Ss*, phospholipase B from *Spilotes sulphureus*; PLB_*Ns*, phospholipase B from *Notechis scutatus*; 4BWC, phospholipase B like protein 1 from bovine kidneys; 3FGW, 66.3 kDa protein from *M. musculus*. The amino acid residues involved in catalysis are underlined with blue, and the cysteine residues are underlined with yellow. The consensus lipase sequence is underlined with gold color. The cysteine residues that make disulfide bridges are linked (yellow lines). The putative *N*-glycosylation amino acid residues are underlined in green. The secondary structure elements (α-helices and β-strands) are shown above the sequence.

The primary amino acid sequence of PLB_*Bm* contains 553 amino acid residues in the precursor form and 491 amino acid residues in the mature form (Amorim et al., 2017). The sequence alignment analysis indicates a high sequence identity (70–97%) among SVPLBs, moderate sequence identity (63–67%) with cow PLB, and very low sequence identity (34%) with mouse PLB (Figure 1 and Table 2). The primary amino acid sequence of PLB_*Bm* contains seven cysteine residues in the precursor form and five cysteine residues in the mature form (Figure 1). Of the five cysteine residues, four make two disulfide bonds (Cys88–Cys500 and Cys499–Cys523), whereas one cysteine (Cys237) remains in the free form. This free cysteine functions as one of the main amino acids in the active site of these enzymes, and they are also called cysteine proteinases (Verma et al., 2016). This cysteine residue is fully conserved in all SVPLBs and also PLBs of cow and mouse (Figure 1). The four other cysteine residues are fully conserved among SVPLB and mouse PLB; however, in the cow PLB, the cysteine at positions 501, 502, and 523 are not conserved (Figure 1). The amino acid residues belonging to the active sites (Asp303, Lys527, Cys237, His254, and Arg265) are also fully conserved among SVPLBs and mouse and cow PLBs. SVPLBs contain one glycosylation site (Asn69), which is fully conserved with the mouse PLB. The concurrence (consensus) lipase sequence GXSXG is fully conserved among all the aligned PLBs (Figure 1). The analysis of the phylogenetic tree generated from the aligned sequence shows a close relationship among SVPLBs and PLBs from mouse and bovine kidneys (Supplementary Figure S1).

**TABLE 2:** Percent sequence identity among snake venom PLBs, PLB-like protein 1 from bovine kidneys, and 66.3 kDa protein from *Mus musculus*.

Proteins	PLB_*Bm*	PLB_*Ca*	PLB_*Oo*	PLB_*Pf*	PLB_*Ss*	PLB_*Ns*	PLB_*Cj*	4BWC	3FGW
PLB_*Bm*	–	96.93	96.20	95.84	87.70	84.45	70.58	63.57	34.33
PLB_*Ca*	96.93	–	96.56	96.93	88.79	86.26	70.95	62.98	34.40
PLB_*Oo*	96.20	96.56	–	95.48	87.16	84.99	71.32	63.37	34.24
PLB_*Pf*	95.84	96.93	95.48	–	89.33	85.71	72.63	64.15	33.65
PLB_*Ss*	87.70	88.79	87.16	89.33	–	96.60	72.02	64.73	34.02
PLB_*Ns*	84.45	86.26	84.99	85.71	96.60	–	64.73	62.40	33.65
PLB_*Cj*	70.58	70.95	71.32	72.63	72.02	70.40	–	67.57	34.19
4BWC	63.57	62.98	63.37	64.15	64.73	62.40	67.57	–	34.46
3FGW	34.33	34.40	34.24	33.65	34.02	33.65	34.19	34.46	–

*PLBs, phospholipases B; PLB_Bm, phospholipase B from Bothrops moojeni; PLB_Pf, phospholipase B from Protobothrops flavoviridis; PLB_Ca, phospholipase B from Crotalus atrox; PLB_Oo, phospholipase B from Ovophis okinavensis; PLB_Cj, phospholipase B from Coturnix japonica; PLB_Ss, phospholipase B from Spilotes sulphureus; PLB_Ns, phospholipase B from Notechis scutatus; 4BWC, phospholipase B like protein 1 from bovine kidneys; 3FGW, 66.3 kDa protein from M. musculus.*

### Domain Analysis

The ThreaDom (Threading-based Protein Domain Prediction) (Xue et al., 2013) analysis indicates that PLB_*Bm* is a single-domain protein. The molecular weights (calculated through ProtParam (Gasteiger et al., 2005) of the precursor and mature protein were 63.88 and 57.09 kDa with the corresponding p*I* of 8.80 and 7.71, respectively. These results indicate that the p*I* of PLB_*Bm* changes from highly basic to slightly basic upon maturation. The theoretically calculated molecular weights and p*I*s agree with the experimentally observed molecular weights and p*I*s of these enzymes (Doery and Pearson, 1964; Takasaki and Tamiya, 1982; Bernheimer et al., 1986, 1987; Chatrath et al., 2011).

### Homology Modeling

For the 3D structure characterization of PLB_*Bm*, the homology model was generated using the online modeling servers like the SWISS Model (Waterhouse et al., 2018), I-TESSER (Laskowski et al., 2001), and MODELLER 9v19 program (Webb and Sali, 2016). The atomic coordinates of phospholipase B-like protein 1 from *Bos taurus* (PDB ID: 4BWC; 70% amino acid sequence identity with PLB_*Bm*) (Repo et al., 2014), were applied as a template.

### Model Validation

The generated model of PLB_*Bm* was validated using programs like PROCHECK, ERRAT, and Verif3D software (Bowie et al., 1991; Lüthy et al., 1992; Colovos and Yeates, 1993; Laskowski et al., 1993). The best model was selected based on the analysis coming from these programs. The PROCHECK analysis of the best 3D structure model of PLB_*Bm* shows that 95.7% (468 amino acid residues) were in the favored region and 4.3% (21 amino acid residues) were in the allowed region with no amino acid residue in the outlier region of the Ramachandran plot (Lovell et al., 2003; Supplementary Figure S2). The overall quality factor of the ERRAT analysis was 96 (Supplementary Figure S3), which lies for the best structure quality of the proteins 3D structure according to the writers of the program (Colovos and Yeates, 1993).

### Molecular Dynamics Simulation

The programs used for the molecular dynamics (MD) simulation includes GROMACS (Berendsen et al., 1995; Maier et al., 2015), AMBER16 (Case et al., 2005; Salomon-Ferrer et al., 2013), MDWeb, and MDMobby (Hospital et al., 2012). The analysis of the MD simulation coming from all these programs indicates the same results for the modeled structure of PLB_*Bm* (Supplementary Figures S4A–D). The important 3D structure parameters like chirality, disulfide bonds, and unusual *cis*/*trans* configuration were correct, and there were no steric clashes in the modeled PLB_*Bm* structure (Supplementary Figure S4A). The analysis of the root-mean-square deviation (RMSD) and radius of gyration (RG), the two essential parameters for 3D structure validation, have shown that the PLB_*Bm* has not undergone substantial changes during the modeling process. The RMSD value did not diverge more than 1 Å (Supplementary Figure S4B), and the radius of gyration was constant (kept around 21.5 Å) throughout the MD simulation process (Supplementary Figure S4C). The B-factor per residue was ∼17 Å (Supplementary Figure S4D), which lies in the average B-factor range for the proteins with X-ray resolution (1.8–2.1 Å) (Carugo, 2018).

### The Overall Structure of Snake Venom Phospholipase B

**FIGURE 2:**
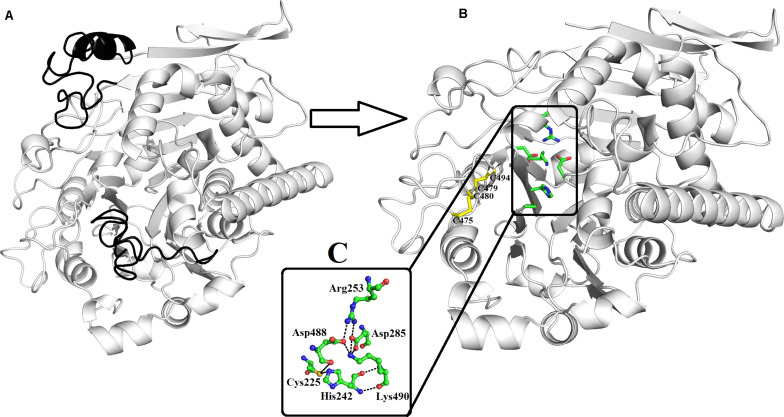
Overall structure of phospholipase B from *Bothrops moojeni* (PLB_*Bm*) cartoon representation of **(A)** zymogen and **(B)** mature protein. The signal and the inner peptides are shown in black color. The active site residues and the disulfide bridges are shown as green and yellow sticks. **(C)** The residues involved in catalysis are highlighted.

The mature protein of PLB_*Bm* contains 491 amino acid residues that fold into a well-defined 3D structure, which contains four-layer αββα sandwich core and has a typical fold of the N-terminal nucleophile aminohydrolase (Ntn-hydrolase) (Figures 2A,B; Oinonen and Rouvinen, 2000; Lakomek et al., 2009; Repo et al., 2014). The overall structure of PLB_*Bm* contains 18 β-strands and 17 α-helices with many connecting loops (Figures 2A,B and Supplementary Figure S5). The structure divides into two chains (A and B) after maturation (Oinonen and Rouvinen, 2000; Repo et al., 2014). The A chain is small and contains 207 amino acid residues, whereas the B chain is large and contains 266 amino acid residues (Supplementary Figure S5).

The A chain has four β-strands numbered 1 through 4 and five α-helices named A to E (Supplementary Figure S5). The β-strands are anti-parallel to each other. The N-terminal of this chain starts with long β-strands, and the C-terminal ends at α-helix (Figure 2B and Supplementary Figure S5). This chain is stabilized by four intrachain salt bridges (His110–Asp69, Arg144–Asp167, Arg144–Asp140, and Arg159–Asp55) and two interchain salt bridges (Lys82–Asp259 and Arg157–Asp264) (Table 3).

**TABLE 3:** Salt bridges in the PLB_*Bm* three-dimensional structure.

Residue 1	Residue 2	Distance
NZ LYS A 82	OD2 ASP B 259	3.51
NE2 HIS A 110	OD2 ASP A 69	3.89
NH1 ARG A 144	OD2 ASP A 167	2.87
NH2 ARG A 144	OD2 ASP A 140	3.43
NH2 ARG A 157	OD2 ASP B 264	3.65
NH1 ARG A 179	OD2 ASP A 55	3.59
NH1 ARG B 253	OD2 ASP B 285	3.94
NH1 ARG B 253	OD2 ASP B 488	2.83
NZ LYS B 358	OD2 ASP B 356	3.59
NZ LYS B 382	OD1 ASP B 403	2.84
NZ LYS B 382	OE1 GLU B 405	2.90
NH2 ARG B 391	OD1 ASP B 368	2.72
NH1 ARG B 436	OD1 ASP B 459	2.74
NH2 ARG B 436	OD2 ASP B 437	2.76
NH2 ARG B 436	OD2 ASP B 459	2.70
NZ LYS B 440	OD1 ASP B 437	2.68
NH2 ARG B 473	OD1 ASP B 475	3.77
NH2 ARG B 479	OD1 ASP B 477	3.12
NZ LYS B 490	OD2 ASP B 285	2.93
NZ LYS B 490	OD1 ASP B 488	2.80

*PLB_Bm, phospholipase B from Bothrops moojeni.*

The B chain is more compact, and it contains 14 β-strands and 12 α-helices. Of the 14 β-strands, seven are parallel, whereas the other seven are antiparallel. The N-terminal of this chain starts with a long β-strand, and the C-terminal ends with a short β-strand. The active site is located in this chain (Figure 2B). This chain is stabilized by two interchain salt bridges (Lys82–Asp259 and Arg157–Asp264) and 14 intrachain salt bridges (Arg253–Asp285, Arg253–Asp488, Lys358–Asp356, Lys382–Asp403, Lys382–Glu405, Arg391–Asp368, Arg436–Asp459, Lys440–Asp437, Arg473–Asp475, Arg479–Asp477, Lys490–Asp285, and Lys490–Asp488). This chain is further stabilized by two intrachain disulfide bridges (Cys88–Cys500 and Cys499–Cys523) (Figures 1, 2B).

### Active Site

A 16-amino-acid-residue segment (208–224) is removed autocatalytically between chains A and B, which opens the active site and produces a cavity that facilitates the entry of a substrate to the active site (Figure 2B). The free cysteine residue (Cys225), which is situated between the key β-sheets in chain B, forms the active site of this enzyme. This cysteine residue functions as both a nucleophile and a general base during catalysis. It is further supported by His242 and Lys490, which is in turn assisted by Asp285, Asp488, and Arg253 (Figure 2C). These active site residues are conserved in the PLBs from other organisms as well (Figure 1 and Supplementary Figure S6). The sequence logo produced from the aligned sequence of SVPLBs and PLBs of mouse and bovine kidneys display high sequence identity around the active site (Supplementary Figure S7).

### Glycosylation

PLB_*Bm* contains carbohydrate moiety like PLBs from other organisms (Lakomek et al., 2009; Repo et al., 2014). The NetNGlyc server1 (Gupta et al., 2004) found a single glycosylation site for this enzyme at Asn69. In the primary amino acid structure of *Drysdalia coronoides* PLB, two putative glycosylation sites have been found (Chatrath et al., 2011). In bovine lysosomal phospholipase B-like protein (PDB ID: 4WBC) (Repo et al., 2014), six glycosylation sites were identified, which include Asn68, Asn211, Asn305, Asn363, Asn408, and Asn523. Of these, only Asn69 that is conserved between PLB_*Bm* and bovine lysosomal phospholipase B-like protein contains *N*-acetylglucosamine (NAG). Although Asn211, Asn305, Asn408, and Asn523 are conserved with PLB_*Bm*, these were found without carbohydrate moiety. In the structure of lysosomal 66.3 kDa protein from mouse (PDB ID: 3FGR) (Lakomek et al., 2009), seven NAG molecules were found, which were attached to Asn93, Asn236, and Asn520 (one NAG each) and Asn115 and Asn441 (two NAGs each). Only Asn93, which corresponds to Asn69 of PLB_*Bm*, is conserved between the two proteins and contains NAG. In SVPLBs, the specific function of the glycan moiety is not fully known; however, it may help the enzyme to specifically bind to the cell surface, thereby facilitating the hydrolysis processes.

### Substrate Specificity

SVPLBs have been shown to hydrolyze phosphatidylcholine, phosphatidylethanolamine, and lysophosphatidylcholine, however, they are not active against phosphatidylinositol, phosphatidylserine, sphingomyelin, and cardiolipin (Supplementary Figure S8; Bernheimer et al., 1986, 1987).

**FIGURE 3:**
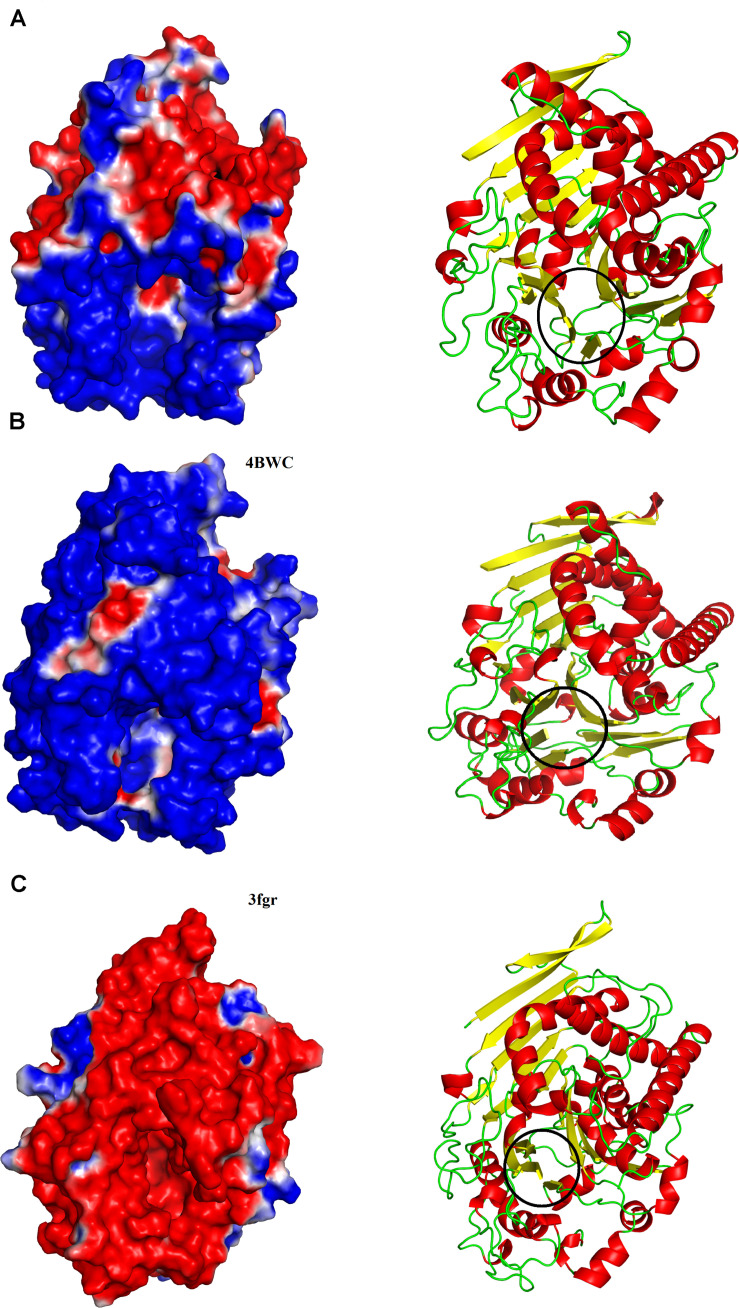
Surface charge distributions of **(A)** phospholipase B from Bothrops moojeni (PLB_Bm), **(B)** phospholipase B like protein 1 from bovine kidneys, and **(C)** 66.3 kDa protein from Mus musculus highlighting the contrasts at the catalytic interface. Black circles indicate the location of the active-site pocket. Blue, red, and white represent positive, negative, and neutral charges, respectively.

On the other hand, PLBs of fungi, bacteria, and mammals have been shown to hydrolyze a broad range of substrates like phosphatidylcholine, phosphatidylinositol, phosphatidylserine, phosphatidylethanolamine, phosphatidic acid, lysophosphatidyl- choline, and lysophosphatidylethanolamine (Supplementary Figure S8; Morgan et al., 2004; Lakomek et al., 2009; Repo et al., 2014). An explanation for the observed specificity of SVPLBs and PLBs from other organisms can be made on the basis of surface charge distribution around the active site cavity (Ullah et al., 2018,2019; Ullah, 2020). In SVPLBs, the active site cavity is negatively charged, whereas the entry to the active site is positively charged (Figure 3A). In the case of bovine lysosomal phospholipase B-like protein, the active site cavity and its entrance are both neutral and positively charged, respectively (Figure 3B), whereas lysosomal 66.3 kDa protein from mouse that is also a PLB has an active site cavity that is highly negatively charged, and its entrance is also negatively charged (Figure 3C). A second factor that may involve this substrate specificity is the volume of the active site cavity. The SVPLBs have large active site cavity volumes with long average depth (Table 4). Owing to this, the phospholipids with the large polar head group easily reach their active sites, whereas the PLBs from other organisms have relatively small cavity volume with small depth and can accommodate phospholipids with a small polar head group (Table 4). The size of the active site cavity gradually decreases in PLB_*Bm* while going from the surface to the interior of the protein (Figure 3A). From the above discussion, it is clear that the enzymes showing specificity for phosphatidylcholine, phosphatidylethanolamine, and lysophosphatidylcholine (SVPLBs) have negatively charged active site with a large volume, which can accommodate the phospholipids with large and positively charged head groups (Supplementary Figure S8). The other PLBs (fungi, bacteria, and mammals) having broad specificity display surface charge distribution (partially positive and neutral and highly negative), and the active site with a relatively small volume can accommodate phospholipids with head group that is positively and negatively charged or neutral (Supplementary Figure S8).

**TABLE 4:** Average active site cavity volumes and average active site cavity depths of PLB_*Bm* and their mammalian counterparts.

Protein	Average volume (Å^3^)	Average depth (Å)
PLB_*Bm* model	5,740.88	15.71
4BWC	2,911.36	11.16
3FGR	4,231.83	13.35

*PLB_Bm, phospholipase B from Bothrops moojeni.*

### Maturation of Snake Venom Phospholipases B

**FIGURE 4:**
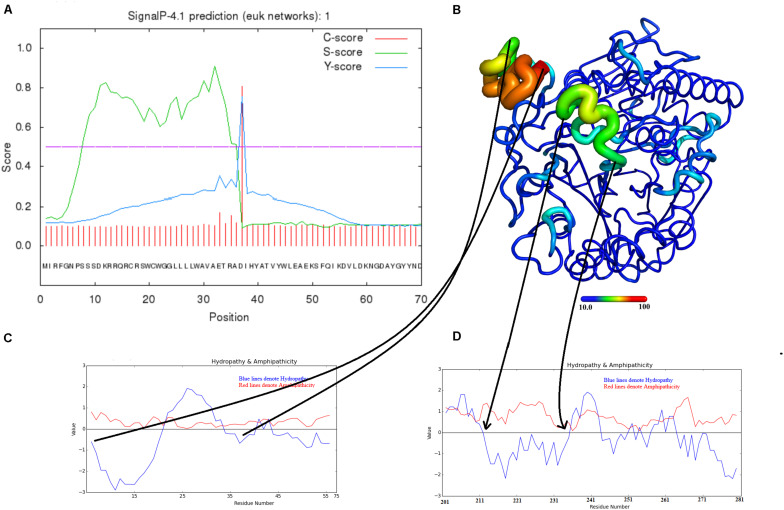
Maturation mechanism for phospholipase B from *Bothrops moojeni* (PLB_*Bm*). **(A)** A signalP-HMM prediction plot, **(B,C)** a Kyte–Doolittle plot for signal and activation peptides, and **(D)** ribbon representation of colored B-factor (temperature)

**FIGURE 5:**
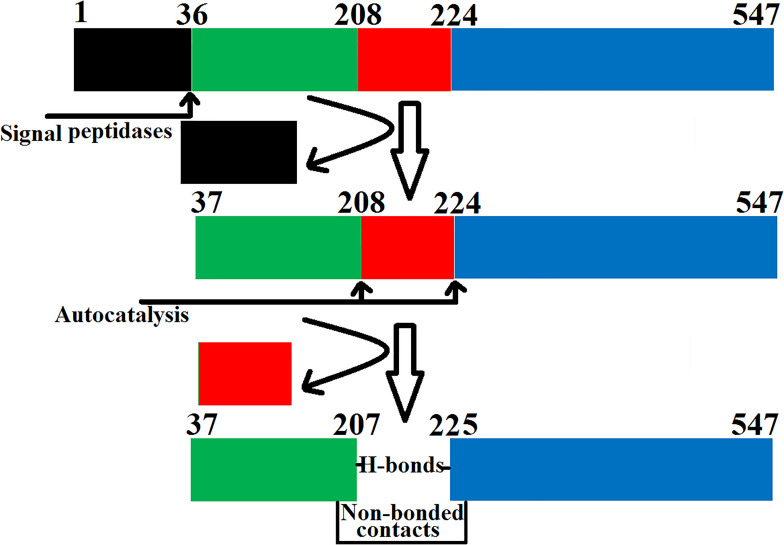
Steps involved in the maturation of phospholipase B from *Bothrops moojeni* (PLB_*Bm*). The prepropeptide of PLB_*Bm* with the signal and internal peptides (colored in black and red, respectively) and the mature protein with chain A (colored in green) and chain B (colored in blue).

The SVPLBs like other snake venom enzymes are secreted as zymogen with the signal peptide, an internal peptide, and a phospholipase domain (Rokyta et al., 2011; Amorim et al., 2017; Figures 4A–D, 5). The zymogen or precursor proteins of SVPLBs contain 547–553 amino acid residues in length (Chatrath et al., 2011; Rokyta et al., 2011; Aird et al., 2017). During the maturation process, the SVPLBs lose the signal peptide. The amino acid sequence analysis by signalP-3.0 (Bendtsen et al., 2004) indicates that this part has 36 amino acid residues (Figure 4A). The signal peptide is removed cotranslationally or by the action of signal peptidases (Paetzel et al., 2002; Figure 5). A second segment (internal peptide) is removed autocatalytically and internally from these enzymes (Oinonen and Rouvinen, 2000). This segment contains 16 amino acid residues (Figure 1). After the removal of the internal peptide, the SVPLBs are divided into two chains like bovine lysosomal phospholipase B-like protein and lysosomal 66.3 kDa proteins from mouse (Lakomek et al., 2009). The two chains are connected by many hydrogen bonds and non-bonded contacts between them (Lakomek et al., 2009). The Kyte–Doolittle plot for hydropathy (Gasteiger et al., 2005) and the temperature B-factor analysis indicate that both the signal and internal peptides are present in the hydrophilic region of the protein (Figures 4B–D).

## Conclusion

• The sequence and structural analysis of PLB_*Bm* was carried out using several computational biology programs.
• The sequence alignment analysis indicates a high sequence identity (70–97%) among SVPLBs, average sequence identity (63–67%) with cow PLB, and very low sequence identity (34%) with mouse PLB.
• The 3D structural analysis of PLB_*Bm* indicates that its structure is composed of four-layer αββα sandwich core and has a typical fold of the Ntn-hydrolases.
• Structural comparisons with PLBs from cow and mouse indicated that the surface charge distribution and the average active site cavity volume and depth vary in these enzymes, which may impart variable substrate specificity to these enzymes.
• The maturation process of PLB_*Bm* involves loss of the signal and internal peptides to convert it into the fully active mature form.
• The structure of PLB_*Bm* described in this work is solely a predicted structure, and these observations need to be confirmed with experimental evidence like X-rays crystallography (Ullah et al., 2020).
• This work will provide a good starting point for future experimental studies of these enzymes.

## Materials and Methods

### Sequence Logo Generated From Multiple Sequence Alignment

The Weblogo 3.2 (Schneider and Stephens, 1990; Crooks et al., 2004) was used to generate the sequence logo from multiple sequence alignment using default parameters.

### Domain Prediction and Biochemical Properties of the PLB_*Bm*

The domain organization and biochemical properties of PLB_*Bm* were predicted using the program ThreaDomEx (Wang et al., 2017) and ProtParam^1^ (Gasteiger et al., 2005), respectively.

### Prediction of Ligand Binding

The ligand-binding sites in PLB_*Bm* were predicted using the 3DLigandSite (Wass et al., 2010) with parameters set to default.

### Prediction of Glycosylation Sites

The ScanProsite tool (De Castro et al., 2006) and NetNGlyc 1.0 Server (Gupta et al., 2004) were used to predict the putative glycosylation sites of PLB_*Bm*. All the parameters were set to default.

### Disulfide Bond Prediction

The disulfide bridges in PLB_*Bm* were checked using the DiANNA webserver (Ferrè and Clote, 2006) and Dinosolve (Darden et al., 1993; DeLano, 2002; Anandakrishnan et al., 2012; Yaseen and Li, 2013; Maier et al., 2015).

### Homology Model Building of PLB_*Bm*

The 3D structure model of PLB_Bm was produced using various protein modeling programs, like I-TESSER (Roy et al., 2010), the MODELLER 9v19 program (Colovos and Yeates, 1993), and the SWISS Model (Waterhouse et al., 2018). The atomic coordinates of Phospholipase B-like Protein 1 from bovine kidneys (PDB ID: 4BWC) that display 70% amino acid sequence identity with PLB_Bm were used as a template (Repo et al., 2014). The best model was carefully chosen based on the quality and validation reports produced by PROCHECK (Webb and Sali, 2016).

### Molecular Dynamics Simulation

The validation of the modeled 3D structure of PLB_*Bm* was carried out through MD simulation using the programs like GROMACS (Berendsen et al., 1995), MDMoby (Hospital et al., 2012), AMBER16 (Maier et al., 2015), and MDweb (Hospital et al., 2012). The FF14SB force field (Darden et al., 1993) was used for all-atom–protein interaction. The protonation states of the amino acid side chain were determined using the web server H^++^ (Anandakrishnan et al., 2012) at pH 7.0. The system was neutralized with chloride ions, was placed in a rectangular box of TIP3P water, and extended to at least 15 Å from any protein atom. The bad contact from the modeled structure was removed by energy minimization of the system for 500 conjugate gradients steps using a constant force constraint of 15 kcal/mol.Å^2^. The gradual heating of the system was carried out from 0 to 300 K for 250 ps with a constant atom number, volume, and temperature (NVT) ensemble. The protein was maintained with a constant force of 10 kcal/mol.Å^2^. The equilibration step was achieved using the constant atom number, pressure, and temperature (NPT) ensemble for 500 ps. The simulation was carried out for 100 ns with a 4-fs time step. The pressure and temperature were kept constant at 1 atm and 300 K, respectively, by Langevin coupling. The particle-mesh Ewald (PME) method (Darden et al., 1993) was used to compute the long-range electrostatic interactions by keeping the cutoff distance of 10 Å to Van der Waals interactions.

### Model Validation

The PROCHECK software (Laskowski et al., 1993, 2001), ERRAT version 2.0 (Colovos and Yeates, 1993), and Verify 3D (Bowie et al., 1991; Lüthy et al., 1992) were used for validation of the built 3D model of PLB_*Bm*.

### Structure Superimposition

The PyMOL molecular graphics visualization program (DeLano, 2002) was used to align the build PLB_*Bm* model to other homologous proteins from the Protein Data Bank.

### Surface Charge Analysis

The PDB2PQR server program (Dolinsky et al., 2004) was used for charge and radius calculations; and the ABPS Tools from PyMOL was used for surface and charge visualization of the protein (DeLano, 2002).

## Data Availability Statement

The raw data supporting the conclusions of this article will be made available by the authors, without undue reservation, to any qualified researcher.

## Author Contributions

AU designed the project and reviewed the manuscript. RM drafted and thoroughly checked it. Both authors contributed to the article and approved the submitted version.

## Conflict of Interest

The authors declare that the research was conducted in the absence of any commercial or financial relationships that could be construed as a potential conflict of interest.

## Footnotes

1 http://web.expasy.org/compute_pi/
