# Supplementary material for: The Sequence and Three-Dimensional Structure Characterization of Snake Venom Phospholipases B
Source: Front Mol Biosci. 2020 Aug 5;7:175. doi: 10.3389/fmolb.2020.00175 (PMC7419708; doi:10.3389/fmolb.2020.00175)
Supplement: Supplementary file 1 [file Data_Sheet_1.pdf]

Supplementary Material

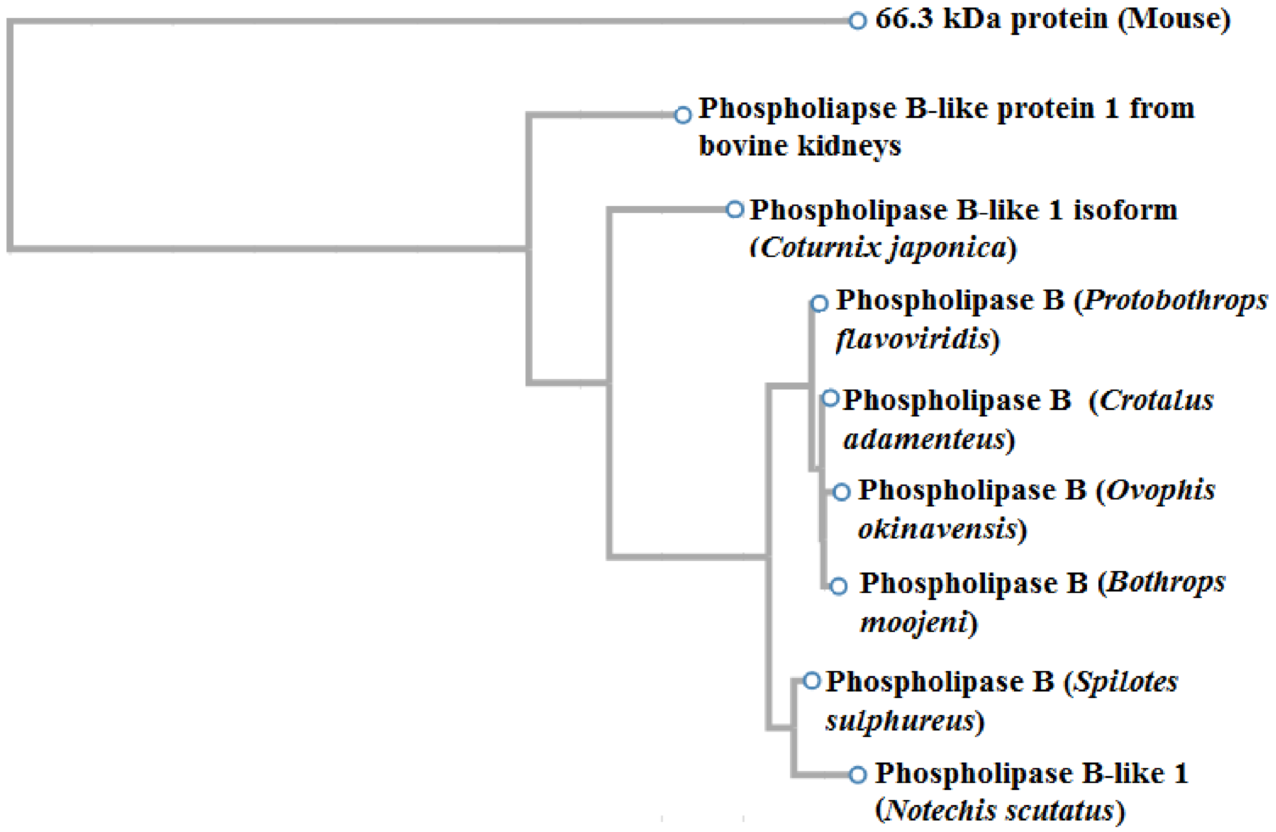

Figure S1: Phylogenetic relationships of PLBs based on protein sequences according to the neighbor-joining method without distance corrections.

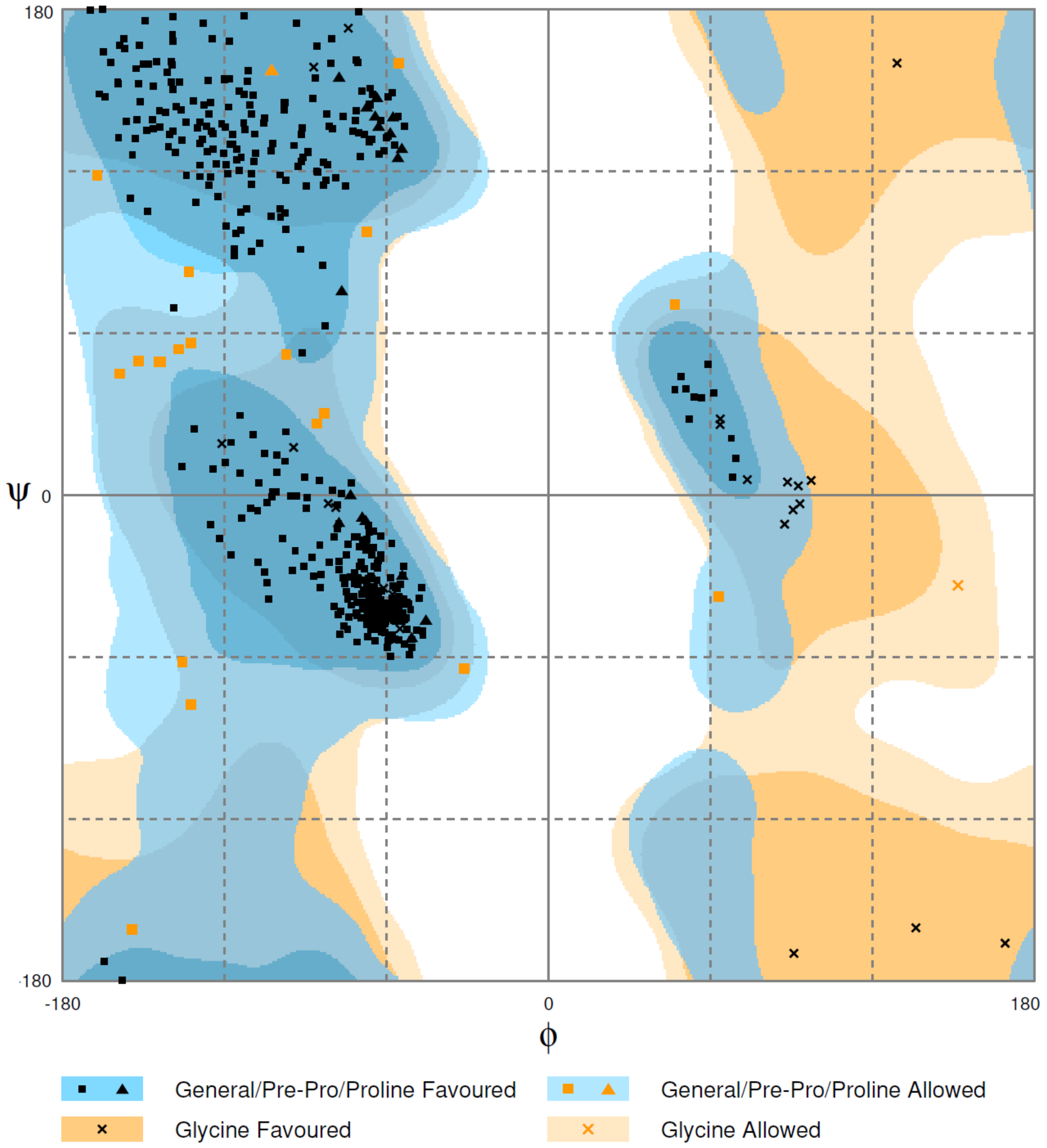

Number of residues in favoured region (~98.0% expected) : 468 (95.7%)
Number of residues in allowed region (~2.0% expected) : 21 (4.3%)
Number of residues in outlier region : 0 (0.0%)

Figure S2: Ramachandran Plot analysis for the build model of PLB_*Bm*.

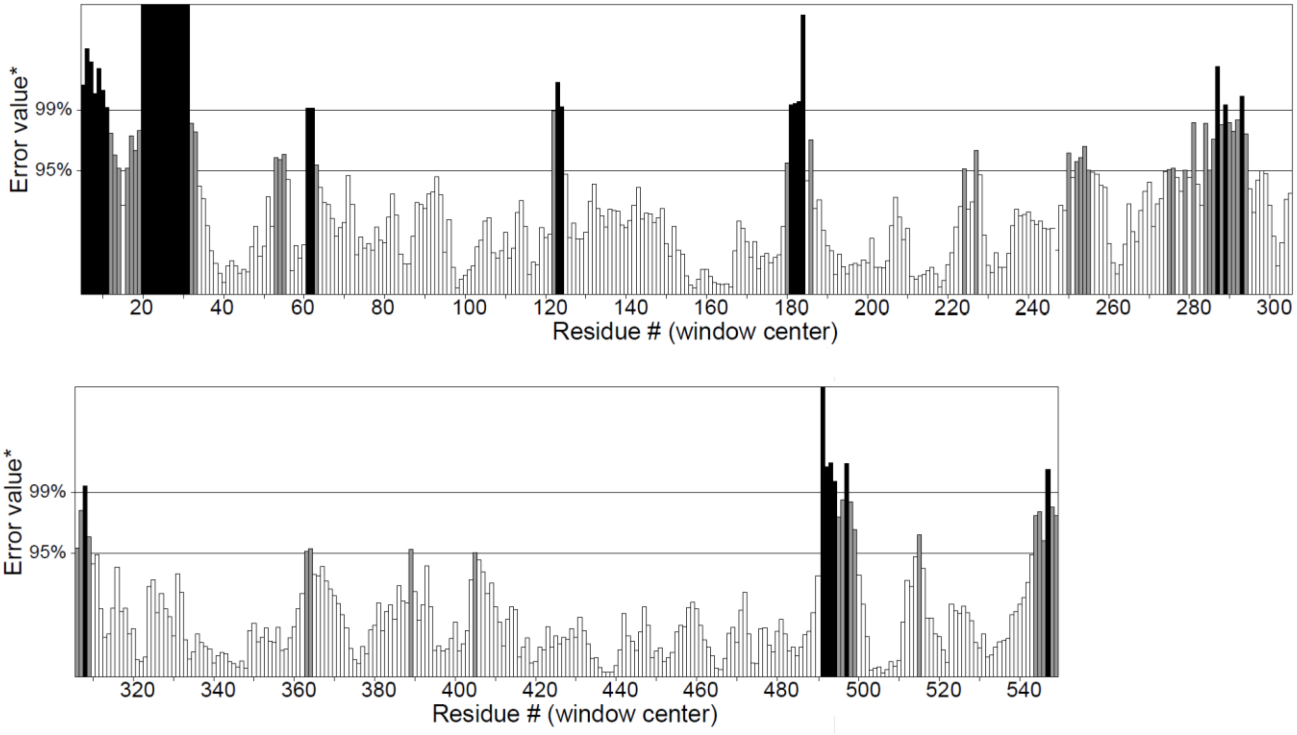

Figure S3: Errors plot for the modeled structure of PLB_*Bm* generated by ERRAT2. The black lines display amino acid residues showing errors.

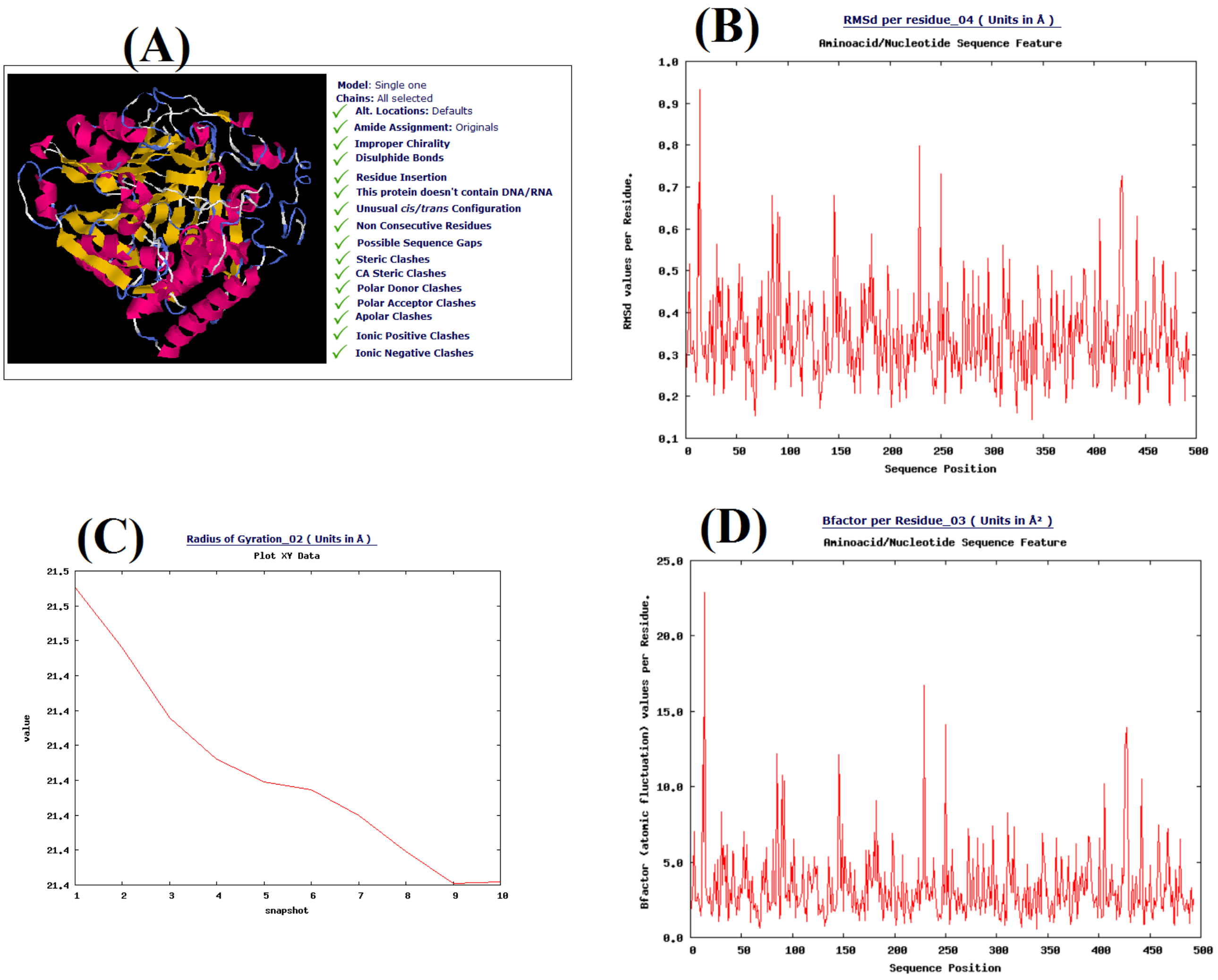

Figure S4: Molecular dynamic simulation analysis of PLB_*Bm* **(A)** Structural parameters **(B)** RMSD per residue **(C)** Radius of Gyration **(D)** B-Factor per residue

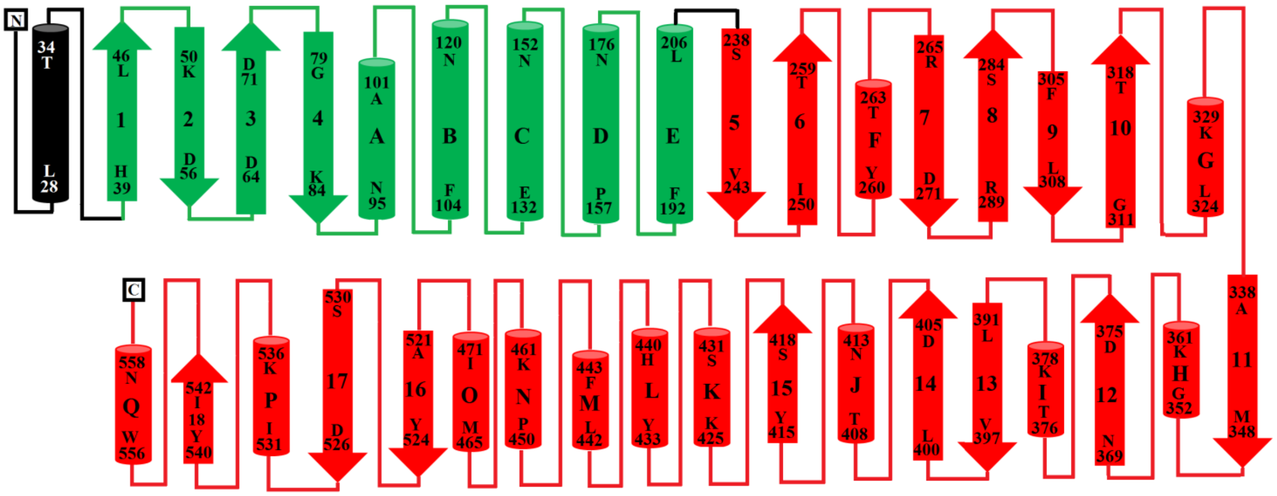

Figure S5: Topology diagram of PLB_*Bm*. The alpha helices (A-Q) and beta strands (1-18) are represented as cylinders and arrows, respectively. Secondary structures and amino acid residues in alpha helices and beta strands were assigned from the primary sequence using the program DSSP and were confirmed with PyMOL from the tertiary structure. Parts of the secondary structure belonging to chain A and B were colored in green and red, respectively.

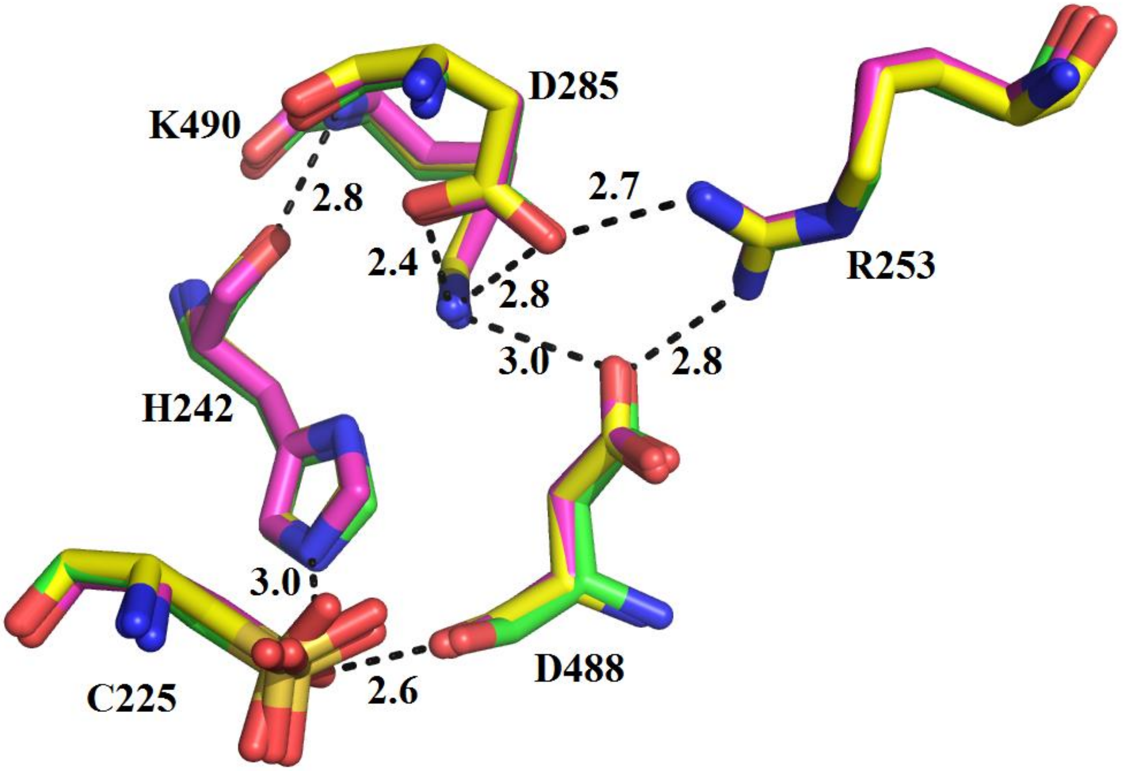

Figure S6: Active site residues of PLB_*Bm* (green sticks), phospholipase B like protein 1 from bovine kidneys (yellow sticks), and 66.3 kDa protein from *Mus musculus* (pink sticks). The Cysteine 225 is shown as OCS (oxidized cysteine).

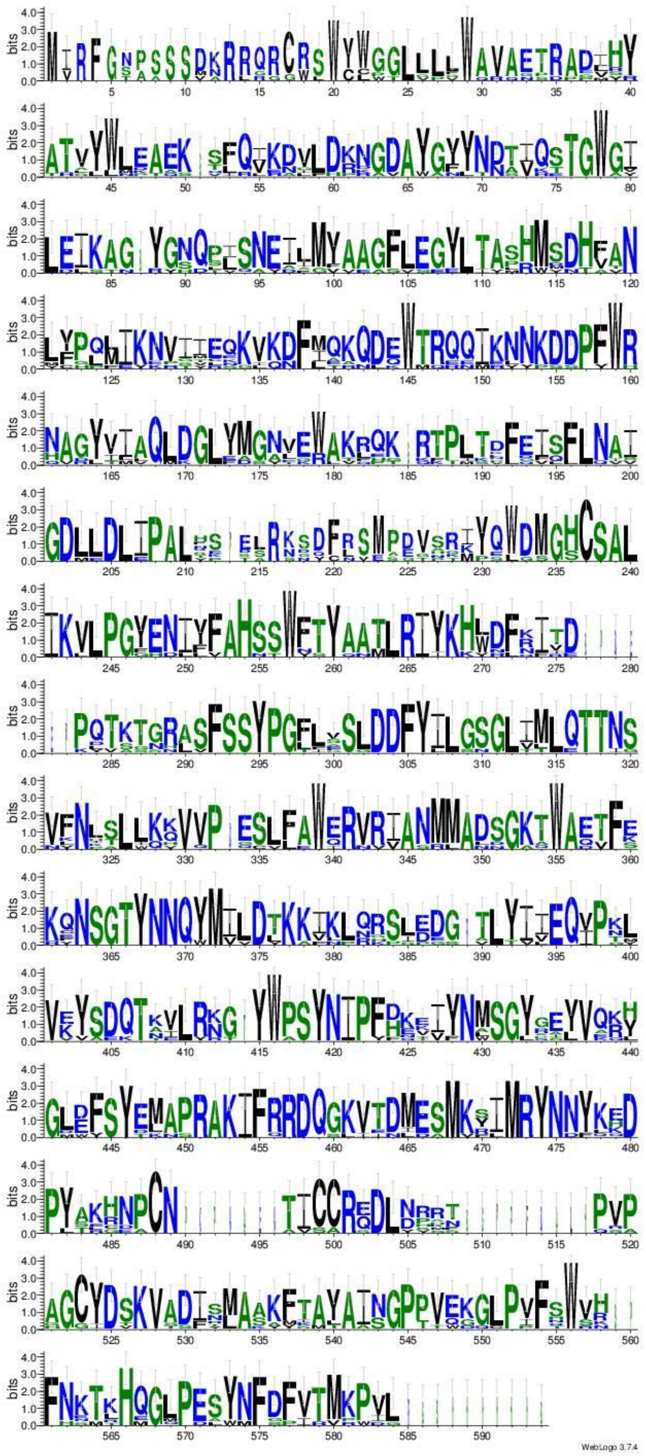

Figure S7: Sequence logo generated from the aligned sequence. The sequence logo displays the most conserved amino acid residues around the active/metal ion binding site from PLBs and their mammalian counterpart.

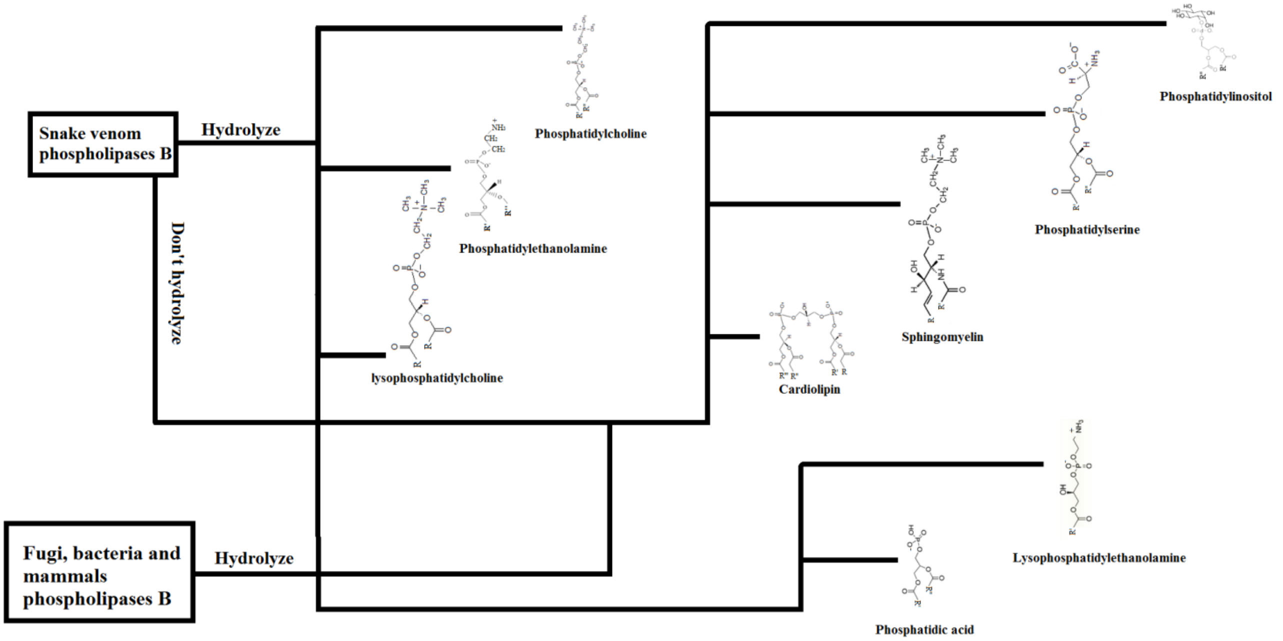

Figure S8: Substrate specificity of SVPLBs and fungi, bacteria and mammals PLBs
